# The effect of integrated care on self-management and emergency department attendance

**DOI:** 10.1192/bjb.2019.1

**Published:** 2019-06

**Authors:** Nikki Scheiner, Sarah Cohen, Ruth Davis, Tim Gale, Amanda Agyare

**Affiliations:** 1 Rapid Access, Interface & Discharge Team (RAID), Hertfordshire Partnership University NHS Foundation Trust, UK; 2 Department of Emergency Medicine, West Hertfordshire NHS Trust, UK; 3 School of Life and Medical Sciences, University of Hertfordshire, UK; 4 Research & Development Department, Hertfordshire Partnership University NHS Foundation Trust, UK

**Keywords:** Frequent attenders, Commissioning for Quality and Innovation, emergency department, Accident and Emergency

## Abstract

**Aims and method:**

The Frequent Attenders Programme is a joint initiative between Hertfordshire Rapid Assessment, Interface and Discharge service and the Emergency Department of the West Hertfordshire NHS Trust, which aims to divert frequent attenders from the emergency department by addressing their unmet needs. This paper describes the range of interventions put in place from the time that the service was set up in 2014 until the introduction of the new national Commissioning for Quality and Innovation 2017–2019, which tasked National Health Service trusts to improve services for people with mental health needs who present to Accident and Emergency. The terms emergency department and Accident and Emergency are used interchangeably, reflecting the practice in policy documents. A subsequent article will report on the impact of the Commissioning for Quality and Innovation in Hertfordshire.

**Results:**

Analysis of the interventions indicated a highly significant (*P* < 0.0001) mean reduction in attendances. Lower gains were made in patients whose primary presentations were alcohol-related. A failure to effect change in two patients led to a significant revision of their respective care plans, resulting in a subsequent reduction in their attendances.

**Clinical implications:**

An integrated approach to patients with complex presentations was associated with high levels of both patient and referrer satisfaction. It is hypothesised that dismantling the barriers between physical and mental health may lead to similar successes in frequent attenders in other in-patient and community medical and psychiatric services.

**Declaration of interest:**

None.

In 2014, there were approximately 21 million visits to hospital emergency departments in England; in 2016, this number had increased to 23.57 million. The increase between 2015 and 2016 was 5.2%, equivalent to an average of 3216 more people attending each day.^1^ Among these, there is a small population of patients who attend on a frequent basis, despite not experiencing a medical emergency. Analysis of the data in Hertfordshire consistently indicates that more than 80% of these patients are either currently open or known to mental health services, a figure that underscores a strong relationship between mental health difficulties, unmet needs and a search for urgent help. The lack of an integrated approach to mental health has been implicated in the significantly reduced life expectancy of those suffering mental illness.^2^

## Rates of frequent attendances to UK emergency departments

The definition of a frequent attender varies, with some National Health Service (NHS) trusts focusing on the admission rate of repeat attenders, and others focusing on the inappropriate attendance rate. In Scotland, a frequent attender is defined as someone who attends the emergency department 10 times or more in a 12-month period, or more than 5 times in a 3-month period. In England, in 2012–2013, 12 000 people made more than 10 visits in a 12-month period to individual emergency departments, with just over 150 attending on more than 50 occasions; some individuals attended nearly 250 times in 1 year.

Official concern about these rates coincide with the release of official figures in early 2015 that recorded the NHS's worst performance in emergency department for a decade, with 8.4% of patients forced to wait for more than 4 h after arriving at hospital. In February 2015, the *London Evening Standard*, using information obtained under the Freedom of Information Act, reported that ‘hundreds’ of patients are visiting London hospitals on more than 20 occasions each year.^3^ In February 2016, the London Ambulance Service disclosed that they currently had 47 768 ‘non-managed’ frequent callers in the capital, resulting in an excess of 183 000 conveyances to hospital.^4^ In June 2017, the *Quarterly Monitoring Report* of The King's Fund, reported that in the previous 12 months, 2,500,000 people in the UK had spent more than 4 h in emergency departments, and that the finance leads of the Clinical Commissioning Groups stated that the pressures on emergency departments are their highest operational concern.^5^

## Hertfordshire initiative to improve care offered to frequent attenders

The awareness of the widespread negative consequences of inappropriate attendances at emergency department led to the creation of The Hertfordshire Rapid Assessment, Interface and Discharge (RAID) Frequent Attenders Programme. This paper describes the development of a joint initiative between Hertfordshire Partnership University NHS Foundation Trust and Watford General Hospital to offer standardised care to frequent attenders. Both the successes and the challenges of implementing individualised and co-produced care plans are discussed, and are followed by reports of the outcome data for the first 2 years of the programme.

The pathway reflected the shared understanding that patients suffering mental health problems face long waits in environments not suited to prevent crisis escalation; many self-discharge before receiving a mental health assessment. Additionally, individuals may face negative attitudes from general hospital staff toward people experiencing a mental health crisis, particularly toward those who self-harm.^6^ Consequently, their needs continue unmet and their attendances persist. At the same time, resources are diverted from patients requiring medical treatment.^1^

## Method

### The West Hertfordshire Frequent Attenders Pathway

#### Aims

**Fig. 1:**
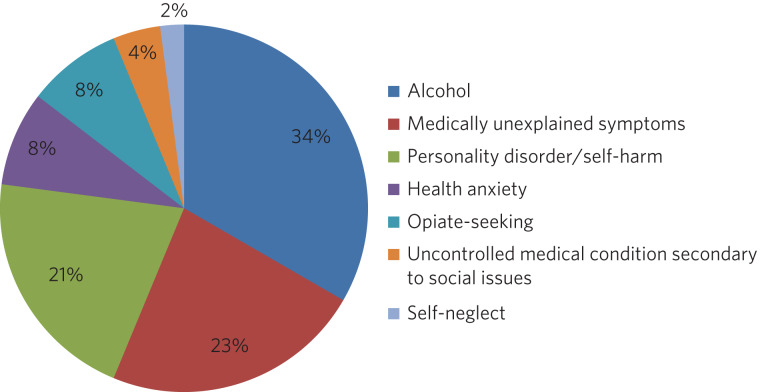
Presenting complaints of frequent attenders to Watford General Hospital.

The West Hertfordshire Frequent Attenders Pathway was set up in early 2014, expanding on an earlier RAID initiative that solely focused on frequent attenders to Watford General Hospital who were also known to mental health service. The revised remit extended to include prolific attenders either not known or not currently open to services. This reflected the finding that in the absence of a bespoke care plan, the frequency of patients' attendances either remained consistent or increased over a 12-month period. A core group comprising the RAID Consultant Psychiatrist and Consultant Psychologist, an Emergency Medicine Consultant and Senior Sister from the Emergency Department at Watford General Hospital and liaison workers from the country drugs and alcohol service Change, Grow, Live, initially met on a monthly basis (now bi-weekly) to identify patients who had attended the emergency department more than 15 times in a 12-month period, or who showed a recent escalating pattern of attendance (Fig. 1: 2009–2013).

#### Method: from single intervention to biopsychosocial assessment and multiagency meetings

Before the 2017–2019 Commissioning for Quality and Innovation (CQUIN), which tasks NHS trusts to achieve a 20% reduction in attendances of patients with mental health difficulties to Accident and Emergency (A&E) departments, referrals were accepted from clinical navigators in the acute general hospital and all staff working in the emergency department and the RAID teams. Cases were prioritised according to clinical need, and individual interventions put in place as appropriate. In some cases, this was as simple as sending an email to a specialty consultant asking for a review of the patient's condition, or involving district nurses in ongoing care of patients' daily needs.

Patients with comorbid physical and mental illness, or only with mental health difficulties, were invited to an assessment with the consultant psychologist. The key features of the assessment were its comprehensive evaluation of all areas of the patient's life (domestic, social, occupational), whether problematic or not, and the lack of time constraint. This enabled the clinician and the patient to achieve a joint understanding of the precipitating and maintaining factors for the frequent emergency department attendances, and formulate a bespoke and dynamic care plan, which could be modified as the patient's circumstances changed.

Patients with more complex presentations, for example, with multiple comorbidities and/or psychosocial difficulties, were discussed at a multiagency meeting, with the patient's general practitioner (GP) playing a pivotal role. To maximise attendance of involved professionals, invitations were sent out 2–3 weeks in advance, and followed up by a telephone call. Meetings were often arranged to take place at the patient's local surgery to accommodate GP clinic schedules; alternatively, conference call facilities were arranged. Participating agencies included the emergency services (the police, the east of England ambulance service, and – on occasion – the fire service), social services, specialty consultants, pain nurses, housing associations, children and family services, community mental health services, service managers and representatives of the two trusts' respective legal departments. In cases when key stakeholders were unable to attend or contribute by telephone, the findings, together with the meeting's draft care plan, were mailed to them.

### The care plan

The patient's and/or carer's involvement in the planning meeting varied between individuals. In cases where guardianship is discussed, family/carers are routinely invited; where there are known engagement difficulties, the patients and their families (if they so wished) were invited to join the second part of the meeting, which would typically be attended by fewer clinicians to reduce potential stress for the patient. Following the agreement with, or at least the acceptance of the draft plan by all stakeholders, it was signed by the patient and a nominated health professional (usually the patient's care coordinator) and then distributed to all agencies and services, including the Mental Health Helpline. A copy of the care plan, including its review date, is kept in the patient's emergency department folder, so that it can be accessed whenever they attend, including out of hours, when junior doctors often come under pressure from patients to provide inappropriate treatments.

A more flexible approach was adopted with patients whose lives tended to be more chaotic often because of psychosocial issues such as homelessness and addiction. The Multi-Disciplinary Team discussion of the needs of these individuals would typically end with an agreement that if they attended the emergency department, the RAID psychologist would be advised so that (if possible) an on-site assessment could be conducted. If this was not possible, the patient would be invited to a biopsychosocial assessment. If the patient did not attend, an attempt was made (with the patient's consent) to meet at their GP surgery. Assessments have also been conducted at a homeless hotel, an intervention dependent upon both the assessor's availability at the time the patient presented and the patient's state of sobriety.

In all cases, emphasis is placed on the therapeutic ethos of the care plan: professionals attempt to balance what is given to the patient (for example, a referral to psychological therapy or access to community activities) with what is taken away from the patient (for example, ambulance conveyance to the A&E department on demand). If the patient's circumstances change, an earlier review meeting may be called.

### Non-engagement

Patient non-engagement with services does not preclude the implementation of a multiagency care plan. It may change, however, the nature of the interventions. *In extremis*, the emergency services (both the police and the ambulance service) imposed a malicious telephony fine for wasting emergency services' time or, on very rare occasions, made an application to the court to demote the security of tenancy or evict a tenant whose frequent calls and troublesome behaviour significantly affected the mental health of vulnerable neighbours. The Frequent Attenders Programme trialled a collaboration with an organisation that worked with those with chaotic lifestyles (typically homelessness and substance misuse) who cost the NHS in excess of £75 000 *per annum*. The organisation, Reducing Chaos, provided transport to patients to support them to attend medical appointments, benefits interviews, addiction groups and meetings relating to their housing.

### Data confidentiality

Terms of Reference for the Frequent Attenders Programme are sent to the Caldicott Guardian of all participating agencies and services to enable the sharing of information on a need-to-know basis.

## Results

In demographic terms, the largest number of frequent attenders were women in the 26–39 years age group. In terms of cost to the NHS, the most expensive group were the over 65 years age group of both genders. These patients typically lived alone, had limited social networks and appeared to derive considerable comfort from the care and attention they received in the emergency department. Young adults (18–25 years) were disproportionately represented in the cohort, highlighting both the lack of good transition services between child and adult mental health services, and the need for improvement in treatments for those with emerging personality disorders.

In the first 2 years of the Frequent Attender Programme, 126 patients were referred to the Frequent Attender Pathway, 90 of whom were deemed appropriate. Of the 36 not accepted, 12 fell below the threshold rate for inclusion on the Pathway; 24 patients reduced their attendances before an intervention was put in place, reflecting an improvement in their housing status. The number includes some out-of-area patients and a small cohort not been previously known to mental health services. Notwithstanding, the majority (>70%) are or have been open to community mental health teams in the county. As expected, most of these patients present with complex needs, including dual diagnosis (mental illness and substance misuse) or dual diagnosis together with a physical health problems. The primary diagnosis of the Hertfordshire frequent attender population is represented in Fig. 1, although it should be emphasised that there is considerable overlap between most of the conditions.

Results for the first 40 patients to complete 12 months after the RAID intervention, whether in the form of a biopsychosocial assessment and simple care plan for a new frequent attender or a multidisciplinary care plan for a patient with chronic and more complex needs, showed a substantial reduction in attendances for just under 90% of patients. In the 12 months pre-intervention, the mean number of attendances was 19.9 (mean, 19.88; s.d. 14.49); this figure dropped to a mean of 6 (mean, 6.00; s.d. 9.95) in the 12 months post-intervention (t(40) = 6.32, *P* < 0.0001). The mean associated cost similarly reduced from £7557 (mean, 7557.58; s.d. 5545.79) 12 months pre-intervention to £2097 (mean, 2097.29; s.d. 3904.20) in the 12 months post-intervention (t(40) = 6.12, *P* < 0.0001). The reductions in both attendance and cost are highly significant (*P* < 0.0001).

In terms of attendances, the highest number for any individual in the 12 months before the RAID intervention was 61; this patient has only attended once in the subsequent 24 months (Table 1). In financial terms, the cost of the most expensive patient on the Pathway was £21 567. The average cost of attendance was £354, as compared with a minimal intervention cost of £67.00 (advice only) and the next level of intervention, which costs £87.00 (advice plus painkillers).^2^ In another case, a patient attended 34 times before the care plan and 4 thereafter.

**Table 1:** Patient vignettes

	Vignette 1	Vignette 2	Vignette 3
Presenting factors	45-year-old male malingerer; long forensic history	67-year-old woman with history of childhood sexual abuse and social deprivation made multiple daily calls to ambulance service; asked ambulance crew to stop at Costa on the way to hospital; asked for sandwiches on arrival at A&E	50-year-old man relapsed 2 months after leaving a private alcohol rehabilitation programme; because of the risk he posed to his children, he was obliged to leave the family home, and ended up sleeping on the streets
Rate of attendance	45 attendances throughout A&E departments in South-East England	45 A&E attendances to West Herts	11 A&E attendances to West Herts
Care plan	RAID psychiatrist worked with the police to place him on the Police National Computer, and negotiated a high threshold for detention under section 136 to avoid reinforcing maladaptive behaviours	Following a professionals meeting, and with her agreement, she was rehoused in supported accommodation	Urgent intervention, facilitated by the Hertfordshire FAP, the emergency department Medical Registrar, CGL and the Crisis Team found him a crisis bed in a residential placement, where he completed a community detox with chlordiazepoxide
Outcome and new rate of attendance	One in the 24 months following the plan	Zero in the 24 months following the plan	Zero in the 24 months following the plan

A&E, Accident and Emergency; West Herts, West Hertfordshire hospitals; RAID, Hertfordshire Rapid Assessment, Interface and Discharge service; FAP, Frequent Attenders Programme; CGL, Change, Grow, Live service.

As shown in Fig. 1, alcohol is the primary presenting issue in 34% of referrals. Patients with alcohol dependence often lead chaotic lives and tend to present to the emergency department only when drunk, making a meaningful assessment difficult. They also tend to ‘disappear’ for extended periods of time, either because they move between counties or because they are sentenced to prison sentences, typically for theft of alcohol. Their attendances also trace a different pattern to other frequent attenders: typically, they have cycles of abstinence alternating with cycles of relapse.

## Discussion

Analysis of the results of the Hertfordshire Frequent Attenders Programme underscores that the most effective way of reducing inappropriate attendances and enhancing patient self-management is locating the frequent attender at the centre of the care plan. The Programme demonstrates that close collaboration between RAID and the emergency department, an integrated multiagency approach and a holistic assessment of the patient's needs improve outcomes. Although individualised care plans can, at least in theory, be drawn up by the Multi-Disciplinary Team in the absence of the patient, the patient's involvement, which may range from a brief assessment in the emergency department up to active co-production, is associated with a greater reduction in attendances. In complex cases, co-production demands a high level of flexibility on the part of the RAID team, including the willingness to offer an outreach service if required. Giving the patient the time they need to explain their difficulties is, unsurprisingly, reflected in individual behavioural change.

The Hertfordshire initiative, as well as programmes set up by other NHS mental health trusts, highlights that many patients who are frequent attenders to their hospital emergency departments have received suboptimal care or simply fallen through a gap in service provision. It is clear that the reasons for non-emergency attendances are complex, and multifactorial. Further, mental illness may or may not be involved in patients' presentations: 24% of the frequent attenders referred to the North-West London local CQUIN (2013–2014) were reported to have complex psychiatric morbidities. Geographical variations reflect different socioeconomic demographics and patterns of migration. What emerges equally clearly is the close relationship between patients receiving suboptimal care and the lack of integration both within and between NHS trusts, and between the health and social care systems. The outcome is either silo provision, gaps in care or duplication.

Several factors are implicated in the failure to provide comprehensive integrated care. Separate commissioning arrangements for substance misuse (a common reason for frequent attendance) and mental health result in disjointed service provision for patients. A lack of communication both between agencies, such as social services, the police and the acute general hospital, and between community and acute teams, forestalls any attempt of a seamless service provision. The problems of commissioning and communication (service factors) are exacerbated by the sizeable number of frequent attenders, often with dependency issues, who access urgent care centres or attend hospitals outside their own trusts (patient factors). In the first scenario, they may provide an alias, or simply not give their full details. In the second case, there is, to date, no communication between trusts unless a dedicated and savvy emergency department consultant alerts colleagues in neighbouring trusts. Requests from neighbouring trusts for information related solely to the number of attendances of an individual are often delayed or even lost in information governance systems. It seems highly probable the current figure of the 200 000 annual unscheduled frequent attendances are an underestimation.

It may be significant that frequent attenders typically make a high number of complaints about the medications and investigations offered, and/or their perceived treatment by emergency department staff. Some inappropriately request admission; others self-discharge prematurely. Many are angry; most are lonely. Those with limited psychological insight may project their frustration with their lives on to the emergency department. The experience of the Hertfordshire Frequent Attender Programme underscores that a multidisciplinary and, where appropriate, multiagency approach helps professionals manage their own emotions and enables patients to reflect on their own difficulties and make meaningful change. The strong therapeutic ethos of care-planning and individual interventions deepens stake-holders' understanding of the interplay between the medical, social, psychiatric and biological vulnerabilities of patients, and is reflected in the positive feedback from both patients and referrers. Exploring the reasons for inappropriate attendances at the emergency department in an unhurried manner, with compassion and an absence of judgement, helps identify not only the predisposing and presenting factors in an individual's maladaptive coping strategies, but also the biopsychosocial perpetuating factors. Once the problem has been clarified, it becomes possible to look for solutions.

### Other frequent attender programmes and future directions

Early work with frequent attenders in 2012–2013, undertaken by the West London Mental Health NHS, led to the development of a local CQUIN in Central North-West London. Building on the work undertaken by the West London Mental Health NHS Trust in 2013–2014, a local CQUIN initiative was developed to identify the most frequent attenders in each of the nine A&E departments, and to create a sustainable model to reduce their unscheduled attendances. The most common primary reason for presenting at an A&E department in this cohort of 128 patients was substance misuse and attendant problems (34%), followed by long-term medical conditions, either with or without a comorbid mental health condition (27%). The elderly frail with globally deteriorating physical health accounted for 15%, and the remaining 24% mainly comprised complex psychiatric morbidity.^7^

A smaller pilot project (*N* = 20) targeting frequent attenders in the Royal Bournemouth Hospital in 2013 reported that, ‘in many cases’, medically unexplained symptoms drive unscheduled presentations to the A&E department.^8^ This finding may be contextualised in light of the report by Bermingham *et al* into the cost of somatisation among the working population in England and Wales in 2008–200,9 which found the cost to the Exchequer of patients with medically unexplained symptoms to exceed £18 billion, a figure that may be compared with the cost of caring for people with dementia.^9^ Costs to the NHS (investigations, treatments, consultations) amounted to £3 billion, with the rest being accounted for by benefit payments, loss of productivity through unemployment and reduced quality of life. In the time since the publication of the report, these figures have increased (Senior Policy Advisor, Centre for Mental Health, personal communication, 2016).

Further analysis was provided by Clifford Mann, President of the College of Emergency Medicine, who identified two cohorts of inappropriate frequent attenders at the emergency department: those unwilling to wait for a GP appointment and migrants, unfamiliar with the English system of healthcare.^10^

The initiatives outlined above together with the work in Hertfordshire indicates that the scope for developing the model is considerable. An assertive outreach pathway is already being trialled by the Watford RAID service. The pathway could additionally be extended to include frequent attendances to GPs and to primary and secondary community services, as well as to admissions to the acute general hospital. Integrated commissioning with other projects that target unmanaged complexity and frequent service use is already under discussion. The potential both for sharing good practice, training and education at a local, regional and national level is considerable. Helping people improve their quality of life through self-management, as well as bringing about significant savings to health and social care economies, benefits individuals and the wider society.

### Limitations

The limitations of the study relate to both internal and external validity. The study design does not allow causality to be established between the intervention (the care plan) and the outcome (the frequency of attendances). Aware that the use of a control group or a randomised methodology were both ethically problematic, the researchers compensated by collecting both retrospective and prospective data. Future studies will seek to establish internal validity by increasing the size of the population studied and extending the follow-up period to 3 or 5 years.

The regional focus of the study limits its generalisability. Relative to London, Yorkshire and North-East England, Hertfordshire (and other regions of the East of England) has a small refugee and migrant population. Comparing interventions and outcomes with the results in areas with large populations of asylum seekers and migrants, who typically use the emergency department as their first port of call, will improve the level of evidence, perhaps leading in time to the development of a national protocol for improving the lives of frequent attenders.

## Footnotes

1 Hertfordshire RAID is currently working with the police to harmonise the risks assessments that the NHS and the police use to determine the most appropriate course of action when a patient leaves the emergency department before assessment.

2 Figures do not include unscheduled GP attendance or East of England triage (£97 for ‘hear and treat’; £237 for conveyance). The costs of residential care placements are similarly excluded.

## About the authors

**Dr Nikki Scheiner** is a consultant psychologist in liaison psychiatry with Hertfordshire Partnership University NHS Foundation Trust, UK. She is the founder of the Frequent Attenders Programme, together with Dr Sarah Cohen. **Dr Sarah Cohen** is a consultant psychiatrist now specialising in perinatal medicine with the Hertfordshire Partnership University NHS Foundation Trust, UK. **Dr Ruth Davis** is a consultant in emergency medicine at Watford General Hospital, UK. **Professor Tim Gale** leads the Research and Development Department at Hertfordshire Partnership NHS Foundation Trust and is also a visiting professor at the School of Life and Medical Sciences at the University of Hertfordshire, UK. **Amanda Agyare** is a trainee counselling psychologist with Hertfordshire Partnership University NHS Foundation Trust, UK.
